# Empirical study of lane-changing behavior on three Chinese freeways

**DOI:** 10.1371/journal.pone.0191466

**Published:** 2018-01-24

**Authors:** Mingmin Guo, Zheng Wu, Huibing Zhu

**Affiliations:** 1 Department of Aeronautics and Astronautics, Fudan University, Shanghai, China; 2 Faculty of Architectural, Civil Engineering and Environment, Ningbo University, Ningbo, China; Beihang University, CHINA

## Abstract

Lane-changing (LC) behavior is investigated on Chinese freeways, where the driving circumstances are relatively aggressive. Three data sets were collected from urban expressways and an intercity highway in the form of traffic videos. Different aspects of LC behaviors are analyzed, i.e., the LC rate, motivation, target lane choice and impact on traffic. The results suggest that LC is a transient behavior that randomly occurs with high frequency, which is the main feature of aggressive driving. Several LC patterns and the combination effect of ramps, fast lanes and various vehicle types are presented. The influence of LC on local traffic endures for approximately 15 to 30 s, which rapidly increases and slowly declines. LC behavior will increase the risk of high-speed car-following. All results are obtained from the empirical data; they will be useful for traffic management and traffic modeling.

## Introduction

As a lateral driving behavior, lane-changing (LC) has a substantial impact on traffic operation and safety. Recent studies have revealed the crucial role of LC in traffic breakdown/capacity drop [1,2], traffic oscillation [3], relaxation [4,5], and moving bottleneck [6]. Therefore, characteristics of LC are getting increasing attentions.

Pioneering models that describe the macroscopic LC processes and microscopic LC processes were proposed by Gazis et al. [7] and Gipps [8], respectively. Moridpour et al. [9,10] and Rahman et al. [11,12] reviewed and categorized existing LC models. The concerned factors in these models include motivation, gap acceptance, target lane choice and influence on traffic. A large number of parameters have been introduced to address these factors, which increases the difficulty of calibration and validation. To overcome this problem, Laval et al. proposed a multilane hybrid model that requires only one extra parameter, i.e., the speed difference across lanes [4,6]. Ko et al. jointly considered the speed difference and density difference across lanes to address the viscous effect caused by LC [13]. Jin also proposed a macroscopic model [2,14] based on the famous LWR model [15,16], introducing one extra parameter, i.e., LC intensity. Although these models have concise forms, they cannot address all phenomena in the LC problem [12]. Systematic measures are required [11] to improve the accuracy, fidelity and reliability of LC models.

The empirical data of LC are relatively lacking compared with those of car-following. Chang and Kao performed an empirical investigation using a time-elapsed video recorder [17]. Currently, traffic video is primarily adopted in observing LC behaviors. Among these efforts, the Next Generation Simulation (NGSIM) trajectory data sets [18] are frequently employed. Many researchers have utilized NGSIM data to calibrate and validate their models [2,4,5,14,19,20]. Moridpour et al. applied the data to explore the impact of heavy vehicles on LC [21,22]. However, Knoop et al. noted that the NGSIM data are mostly very congested [23]. Rahman et al. [11] and Zheng [12] both mentioned some downsides of the data. Zheng indicated the danger of over-utilizing NGSIM data, and suggested that the data that contain more diverse driving behaviors, particularly more aggressive driving behavior, is needed [12]. Hidas [24,25] and Zhao et al. [26] proposed new LC types, i.e., the cooperative and the multistep-approach LC respectively, by taking advantage of the traffic videos that were separately collected from Australia and China. These new LC types can capture the vehicular interaction, whereas the previously accepted discretionary/free and mandatory/forced LC cannot capture this interaction. Knoop et al. quantified the number of LCs in free flow from a three-lane freeway equipped with 55 cameras in the Netherlands [27]. They determined that the average LC rate is approximately 0.4 to 0.5 per vehicle kilometer. Lv et al. obtained the relationship between the LC rate and vehicle density based on the traffic videos taken in China [28]. However, the meaning of the units of LC frequency that they adopted (m^-1^min^-1^lane^-1^vehicle^-1^) is not as explicit as the definition provided by Knoop et al. defined [23]. We discovered that the LC is closely relevant to the high-speed-car-following phenomenon using the traffic videos of Chinese highways [29].

In addition to traffic video, other approaches can be employed to measure LC. Knoop et al. determined the number of LCs by splashover effects in loop detector counts [30]. They collected the data from a three-lane freeway in the United Kingdom where the loop detectors are densely placed, obtained the relationship between the LC rates and roadway density, and determined the ramp’s impact on LC. Collection of data from loop detectors is significantly easier than the data collection from video images. However, large errors in this type of data are inevitable because the LC is indirectly determined. Knoop et al. estimated that the error of their method is approximately 10%. Sun and Elefteriadou instrumented a vehicle with digital cameras and GPS and acquired “in-vehicle” data that classified 40 drivers into four general groups according to the LC maneuvers performed on an urban street [31]. A significant benefit of this method is that both the successful LCs and unsuccessful LCs can be recorded, whereas the comprehensive information of ambient traffic is unavailable. Xuan and Coifman also employed an instrumented probe vehicle to monitor the ambient traffic and analyzed the microscopic impacts of LC on traffic flow [32]. However, their observation scope remains limited within a small area before the probe vehicle.

As indicated by this overview, the empirical study of LC is insufficient for providing evidence for modeling and validation. In this paper, we attempt to sort out LC’s characteristics via filed observation, which offers a reference for modeling LC and setting up a benchmark to validate the models. We collected the empirical data from Chinese freeways, where drivers are generally aggressive. These data fill the gap caused by the notion that the majority of existing empirical data were collected from developed countries where good driving behaviors are widespread.

The remainder of the paper is organized as follows: The next section describes the data sets in this study, followed by the methodology employed and detailed analyses of frequency, motivation, target lane choice of LC and its impact on traffic. The last section provides conclusions and remarks.

## Data sets

### Study site

**Fig 1:**
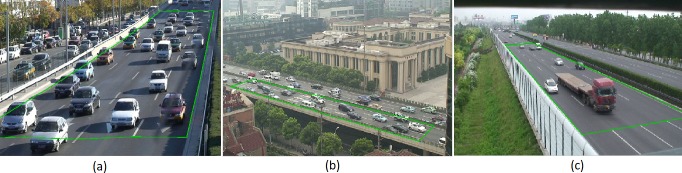
Snapshots of the traffic videos. (a) Section 1. (b) Section 2. (c) Section 3.

From 2008 to 2012, we took more than 300 hours of traffic videos from several urban expressways and intercity highways in China (We have created a website to share these videos and data free of charge: http://traflow.fudan.edu.cn). In this paper, three representative four-lane road sections were selected; they are listed in Table 1. The corresponding snapshots are shown in Fig 1.

**Table 1:** Information of S1, S2 and S3.

Section No.	Freeway type	Location	Shooting time (Weather)	Section length	Ramp
1	Urban expressway	Beijing Forth-Ring Road, west of the Xueyuan Bridge, westbound	2008.11.03 09:05–09:35 (sunny)	100 m	An off-ramp 700 m upstream, an on-ramp 20 m downstream, and another off-ramp 300 m downstream
2	Urban expressway	Yan’an Viaduct, south of the Shanghai Exhibition Center, eastbound	2008.07.21 13:30–14:00 (sunny)	81 m	An on-ramp 250 m upstream and an off-ramp 250 m downstream
3	Intercity highway	Highway G2, west of Shanghai Jiangqiao toll gate, westbound	2012.06.06 07:47–08:17 (sunny)	120 m	An toll gate 1,300 m upstream and an off -ramp 10,500 m downstream

Section 1 and section 2 (abbreviated as S1 and S2) are urban expressways with a speed limit of 80 km/hr. S3 is an intercity highway with a speed limit of 120 km/hr. On the urban expressways, numerous ramps are one or two kilometers or even several hundred meters apart, which severely affects the traffic, especially the LC frequency. S1 is located between an upstream off-ramp and a downstream on-ramp, whereas S2 is located between an upstream on-ramp and a downstream off-ramp. On the intercity highways, the ramps are rather sparse—usually more than tens of kilometers apart, which only affects a small adjacent fraction. S3 is considered to not be affected by a ramp. Few slow vehicles such as trucks use S1 and S2, whereas the proportion of slow vehicles at S3 in our data sets is 9.23%.

### Data collection method

**Fig 2:**
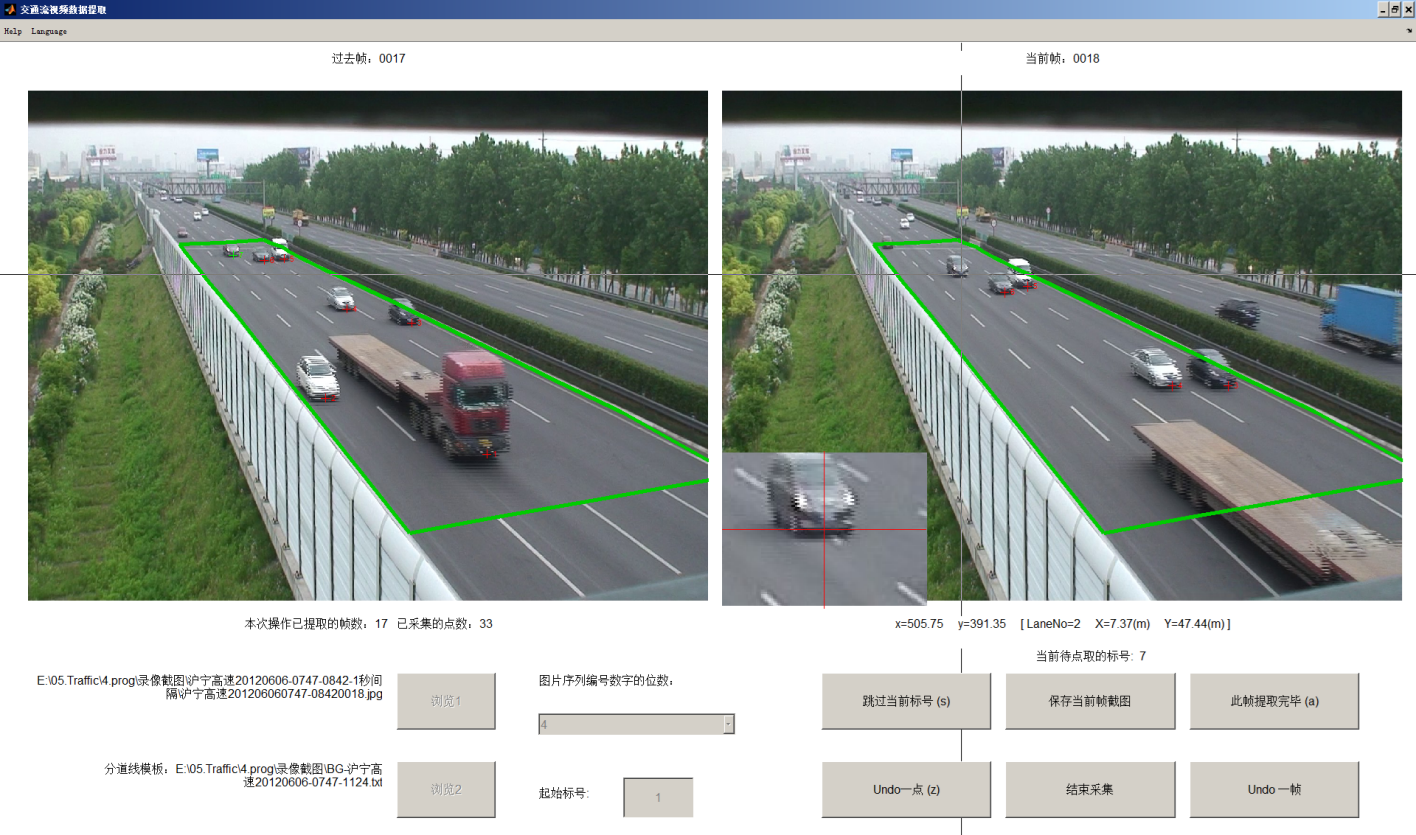
Interactive interface for data collection.

The traffic videos were converted to sequential images with 1-s interval. For each vehicle in the observation scopes (denoted by the green frames in Fig 1), the coordinates in the image of the middle point of the front bumper’s lower edge (close enough to the ground to disregard the projection error) were manually acquired by an interactive interface written with MATLAB, as shown in Fig 2. These coordinates are treated as the representative positions of the vehicles. Given that the length of lane lines (lengths of 6 meters with a spacing of 9 meters) and the width of each lane (3.75 meters) are fixed, the actual position on the road of each vehicle can be obtained by an interpolation transform. Therefore, the vehicle trajectories were collected after all images had been traversed. Sequentially, other parameters such as speed and headway and the occurrences of LC were calculated based on the trajectories.

If the absolute error of position is maintained within 0.5 m, the absolute error of the speed is less than 1.8 km/hr, which is acceptable in this study. The resolutions of our videos are 720 × 576 (at S1 and S2) and 1440 × 1080 (at S3). The distance of 0.5 m usually covers approximately 5 to 10 pixels in the image. As shown in Fig 2, the zoom window can assure this precision. The foreshortening at the distal end of the road, especially at S1 and S3, may amplify the collection error. However, it does not affect the results because this error is limited and these data account for a small part of the total data. The error estimation is detailed in [33].

A complete LC process always requires a certain amount of time, whereas the length of our observation scope is limited. Therefore, we do not care about the detailed LC process. The status of LC vehicles (i.e., the lane changers) is simply divided into pre-LC and post-LC according to whether the middle point of the front bumper crosses the lane line. To prevent the false judgement of LC produced by software, all LCs were confirmed by the videos.

### Data description

All essential data were uploaded as the supporting information in S1 Dataset. In the three excel files, the number, position, speed and headway of each vehicle and numbers of its lead and rear ones in each image frame are provided, based on which various statistical results were obtained.

## Methodology

### Definition of LC rate

A major feature of LC behavior is the frequency of the behavior. Providing a reasonable prediction of the number of LCs is a fundamental criterion to evaluate an LC model. However, the number of LCs varies with the spatial and temporal scale. To perform a comparison, LC rate must be clearly defined. Therefore, we define two parameters, the spatial LC rate *SrLC* and the temporal LC rate *TrLC*, to quantify the average LC rate per unit distance and unit time, respectively.

*SrLC* is defined as

$$SrLC=\frac{N}{Q},$$ (1)

where *N* denotes the number of LCs that occur in 1 hour on a 1 km section of road, with the units of (km•hr)^-1^, and *Q* denotes the flow rate, with the units of veh•hr^-1^. Therefore, *SrLC* has the units of (veh•km)^-1^, which denotes the average number of LCs for a vehicle travelling 1 km. *SrLC* expresses the average probability of the occurrence of 1 LC per vehicle kilometer. Note that the unit of the number of LCs is omitted here.

*TrLC* is defined as

$$TrLC=\frac{N}{K},$$ (2)

Where *K* denotes the density, with the units of veh•km^-1^. Therefore, *TrLC* has the units of (veh•hr)^-1^, which denotes the average number of LCs for a vehicle travelling for 1 hr. *TrLC* expresses the average probability of the occurrence of 1 LC per vehicle hour.

The total number of LCs calculated from the two LC rates should be equal; therefore, we have

SrLC•Q=TrLC•K, (3)

namely,

$$TrLC=SrLC•\bar{v},$$ (4)

where $\bar{v}$ denotes the average vehicle speed.

The formula that directly calculates *SrLC* from the collected data is

$$SrLC=\frac{n}{q}\times \frac{1000}{L},$$ (5)

where *n* denotes the number of observed LCs, *q* denotes the total number of vehicles that pass the road section during the observation, and *L* denotes the road length with the unit of m. *SrLC* and *TrLC* are calculated by Eqs (5) and (4), respectively.

Based on Edie’s generalized definition of flow, density and average speed [34], Knoop et al. also derived several forms of LC rates that are similar to *SrLC* and *TrLC* [23]. However, we do not adopt Edie’s definitions because our data were collected from long road sections with short time intervals. Therefore the definitions of basic traffic parameters in this paper are the same as the definitions provided by Wardrop[34].

According to Eq (3), *SrLC* and *TrLC* are equivalent. Knoop et al. indicates that *SrLC* is the best suitable index to describe the LC frequency [23]. In the analysis of below, *SrLC* was primarily concerned. *TrLC* may be more frequently employed in some microscopic models, therefore, we provided the values of *TrLC* with the units of (veh•s)^-1^.

### Statistical method

#### Relations between LC rate and influential factors

The following factors that affect LC are applied:

- *dv*, speed difference, which is calculated by the speed of the lead vehicle minus the speed of the subject vehicle. *dv*<0 indicates that the subject vehicle moves faster than the lead vehicle.
- *hs*, space headway, which is the distance between the front bumper of the subject vehicle and the front bumper of the lead vehicle.
- *ht*, time headway, which is calculated by dividing the space headway *hs* by the speed.
- *dv*_lane_, speed difference between two adjacent lanes, which is calculated by the average speed of the adjacent lane minus the average speed of the subject lane. *dv*_lane_>0 indicates that the vehicles in the subject lane move slower than those in the adjacent lane.
- *dk*_lane_, density difference between two adjacent lanes, which is calculated by the density of the adjacent lane minus the density of the subject lane. *dk*_lane_<0 indicates that the subject lane has a greater number of vehicles than the adjacent lane.

The relationship of LC rate with respect to one of above variable was obtained as follows: first, for each of the LC vehicles, the corresponding average value of the parameter before the LC occurred was calculated; second, the corresponding average value of the parameter for each of the other vehicles as they pass the road section was calculated; last, based on the respective values, all vehicles were grouped into different intervals of the parameter and the corresponding LC rates were obtained.

#### Principal component analysis (PCA)

We utilized PCA, which is a common statistical method, to investigate the combined effects of the factors. Thus, we determined the primary types of LC patterns. In the PCA, only the LC vehicles were taken into consideration, and *dv*, *hs*, *dv*_lane_ and *dk*_lane_ were standardized by the corresponding mean and standard deviation for multivariate data analysis. The detailed procedures of PCA can be found in many statistics textbooks. In this paper, we performed PCA using the function *pcacov* in Matlab.

## Results and analysis

### LC rate

Table 2 lists the LC rates for each section. At S2, *SRLC* is 1.04 per vehicle kilometer, which indicates that each driver changed lanes averagely more than once per 1 km driven. This rate is approximately one quarter higher than the rate at S1 and S3, i.e., 0.83 per vehicle kilometer and 0.86 per vehicle kilometer, respectively, because the adjacent off-ramp induces additional demands of mandatory LCs in its vicinity.

**Table 2:** LC rates.

Section	Number of vehicles (veh)	Average speed (m/s)	Number of LCs	*SrLC* (veh^-1^•km^-1^)	*TrLC* (veh^-1^•s^-1^)
1	3215	13.07	270	0.8305	0.0109
2	3530	13.55	302	1.0352	0.0140
3	1776	26.13	184	0.8634	0.0226

These results are twice as large of the results reported by Knoop et al. (0.4 to 0.5 per vehicle kilometer) [27]. These LC rates are due to aggressive driving. This type of driver are ready to change lanes in an arbitrary manner at every opportunity. Therefore, a high LC rate is a typical feature of this driving style. S1 Video gives an example of this driving behavior—several LCs at very high speeds and small spacings.

From the perspective of time, the *TrLC* at S3 is twice the *TrLC* at S1, because the spacing between vehicles and average speed on intercity highways are higher than those on urban expressways, which produces a small time interval between two consecutive LCs.

Table 3 lists the lane-specific LC rates that were calculated based on the number of LCs leaving the current lane. The lanes are numbered from left (median, fast) to right (shoulder, slow). At all sections, the LC rates increase from lane 1 (abbreviated as L1) to L3. For L4, the LC rate decreases at S1 and S2 but increases at S3. These phenomena are jointly caused by three types of effects, i.e., anchoring effect, repelling effect and attracting effect, which profoundly affect how the drivers choose lanes.

**Table 3:** Lane-specific LC rates.

Section		Number of vehicles (veh)	Average speed (m/s)	Number of LCs	*SrLC* (veh^-1^•km^-1^)	*TrLC* (veh^-1^•s^-1^)
1	Lane 1	954	14.23	30	0.3145	0.0045
	Lane 2	955	13.82	71	0.7435	0.0103
	Lane 3	879	12.80	103	1.1718	0.0150
	Lane 4	697	11.24	66	0.9469	0.0106
2	Lane 1	862	14.68	41	0.5872	0.0086
	Lane 2	975	13.94	89	1.1269	0.0157
	Lane 3	1046	13.12	122	1.4399	0.0189
	Lane 4	947	12.74	50	0.6518	0.0083
3	Lane 1	612	27.15	32	0.4357	0.0118
	Lane 2	647	26.70	67	0.8630	0.0230
	Lane 3	441	25.28	52	0.9826	0.0248
	Lane 4	260	24.04	33	1.0577	0.0254

The LC rate of L1 is far below the average level at each section, indicating the anchoring effect of the fast (median) lane. Similarly, the anchoring effect of off-ramp makes the LC rate of L4 at S2 significantly decreases with respect to those of L2 and L3.

The repelling effect is attributed to L4 due to an on-ramp or heavy vehicle. At S1, immediately before an on-ramp, the LC rate of L4 is remains as high as 0.9469 veh^-1^•km^-1^. At S3, which is far away from any ramps on an intercity highway, the LC rate of L4 is even higher than the LC rate of L3. Among the 260 vehicles observed on this lane, 74 of the vehicles were slow vehicles such as heavy trucks and 186 of the vehicles were cars. The cars on L4 would usually leave this lane sooner or later to avoid following a slow vehicle and losing speed. Therefore, 28 of the 186 cars changed lanes within our observation scope.

The attracting effect that is caused by the fast lane and off-ramp is primarily reflected on the two middle lanes. Table 4 lists the number of left- and right-turning LCs on the two middle lanes. At S1 and S3, the number of left-turning LCs are greater than the number of right-turning LCs, whereas it is reversed at S2, which is in the vicinity of an off-ramp. The on-ramps and off-ramps are located at the same side of a freeway, therefore the number of left- and right-turning LCs for a vehicle from entering the freeway to leaving the freeway should be equal. For a local road section, the left- and right-turning LCs are unbalanced. The right-turning LCs intensively occur near the off-ramps due to the attraction of the off-ramp; some aggressive drivers would like to change to the shoulder lane as late as possible to maintain the speed. In other places, the left-turning LCs dominate due to the attraction of the fast lane and the repulsion of the on-ramps, which is the cause of density inversion. Note that either left-turning LCs or right-turning LCs are both remarkable anywhere, because that the majority of the LCs are made to achieve the temporary benefit of speed or spacing without a long-term perspective. This type of driving behavior causes high LC frequency and aggressive circumstances.

**Table 4:** Number of left- and right-turning LCs.

Section	Lane 2		Lane 3	
	L1←	→L3	L2←	→L4
1	40	31	56	47
2	32	57	58	64
3	42	25	32	20

### Motivation of LC

A number of factors lead to an LC maneuver. Based on the principle of “vital few and trivial many”, only some vital factors will be analyzed in this section. The mechanism that induces LC is so complicated that an LC maneuver is the result of the nonlinear interaction among these factors. Therefore, the analyses in this subsection can only offer the understandings of LC motivation from different aspects.

#### Effect of speed and speed difference

**Fig 3:**
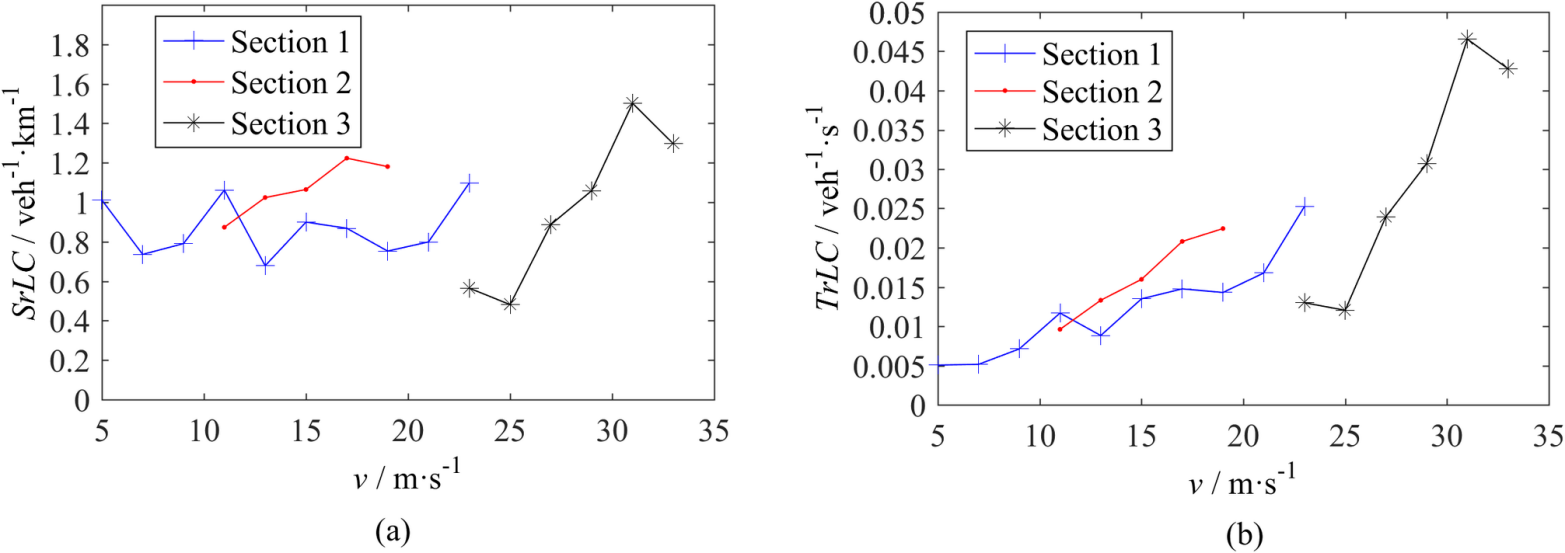
LC rate - speed relationship. (a) Spatial LC rate. (b) Temporal LC rate.

The effect of speed on an urban expressway and intercity highway are different and relevant to the vehicle density. As shown in Fig 3A, at S1 and S2, *SrLC*s almost remain constant with respect to the vehicle speed. The number of LCs of a vehicle within a fixed spatial range has minimal correlation with the speed. A possible reason is that the vehicle density on an urban expressway is relatively high, and therefore, an LC vehicle must move a certain distance to find an opportunity to implement the LC. At S3, *SrLC* increases with an increase in speed because the density here is fairly low and an LC maneuver is easy to implement. Therefore, for aggressive drivers in the condition of high-speed driving, the faster is the speed of a vehicle, the higher is the LC rate. Regarding *TrLC*s, they almost monotonically increase with an increase in speed. The slope of the *TrLC*-speed relationship of S3 is larger than the slopes of the *TrLC*-speed relationship of S1 and S2, as shown in Fig 3B.

**Fig 4:**
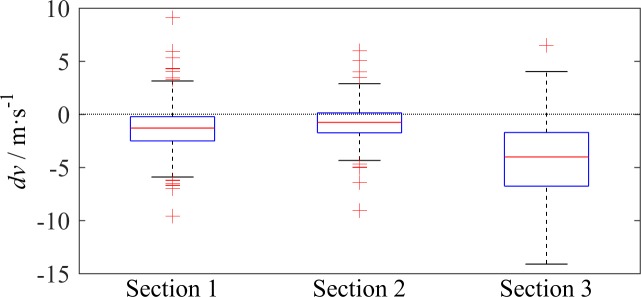
Boxplots of the speed difference of the LC vehicles, the edges of the box are the 25th and 75th percentiles.

**Fig 5:**
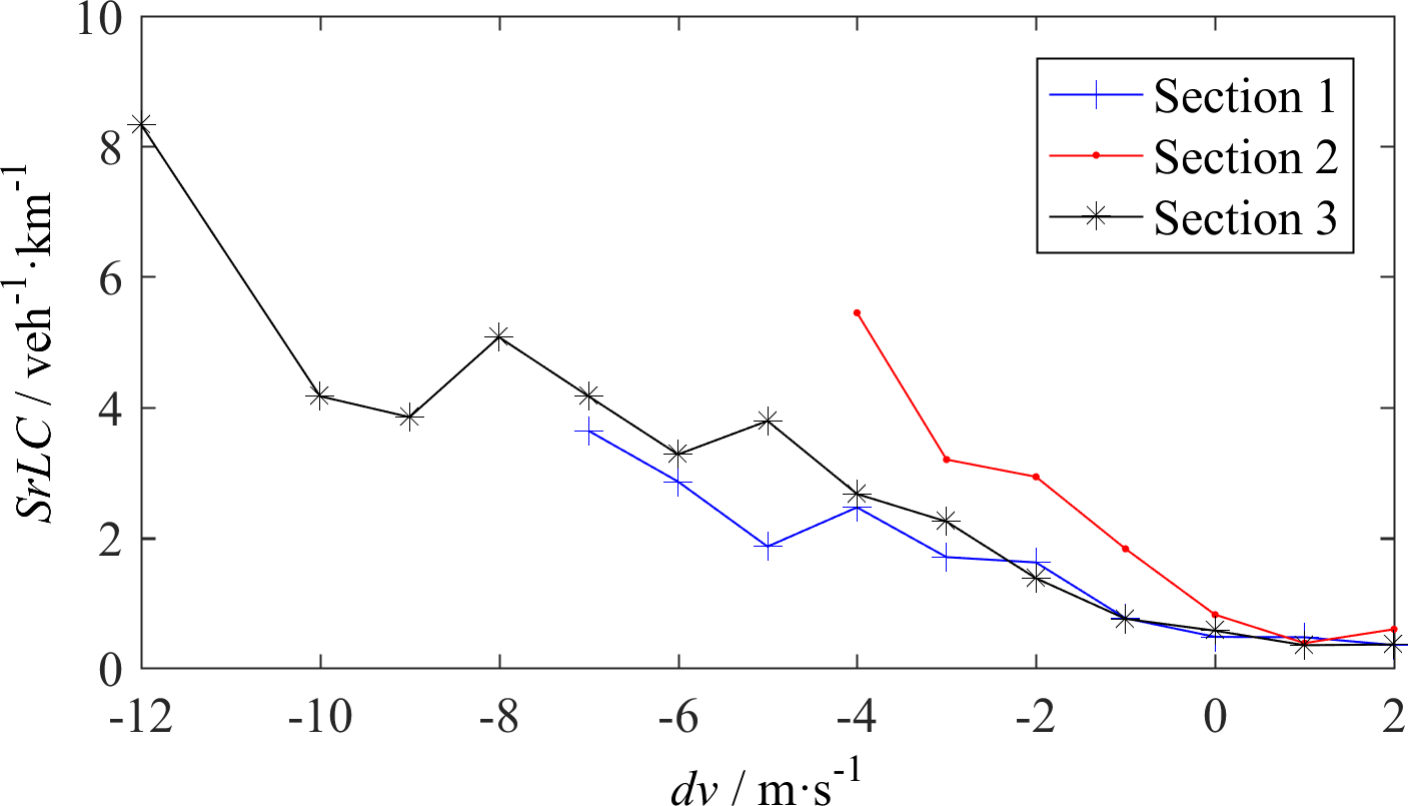
LC rate - speed difference relationship.

The speed difference is usually considered to be one of the prime causes of LC. Fig 4 illustrates the distribution of the *dv* of LC vehicles. Most LCs were triggered by a slow lead vehicle, especially at S3, where 49 of the 184 LC vehicles followed a slow vehicle before changing lanes. Fig 5 reveals the relation between *SrLC* and *dv*. When *dv*<0, as the absolute value of *dv* increases, the *SrLC* almost monotonically increases. The relation between *SrLC* and *dv* can be simply expressed by a linear function, whose slope depends on the location and traffic condition. When *dv*>0, the *SrLC* almost remains constant with respect to *dv* at a certain level, independent of the location and traffic condition. An interesting point in Fig 5 is that *dv* can be as small as -12 m/s at S3, and the maximum corresponding *SrLC* is slightly larger than 8 per veh per km. This result suggests the findings discussed in Subsection 3.1, i.e., the mixture traffic on L4, where the trucks move substantially slower than the cars, will tremendously increase the LC rate in aggressive driving circumstances.

#### Effect of spacing

**Fig 6:**
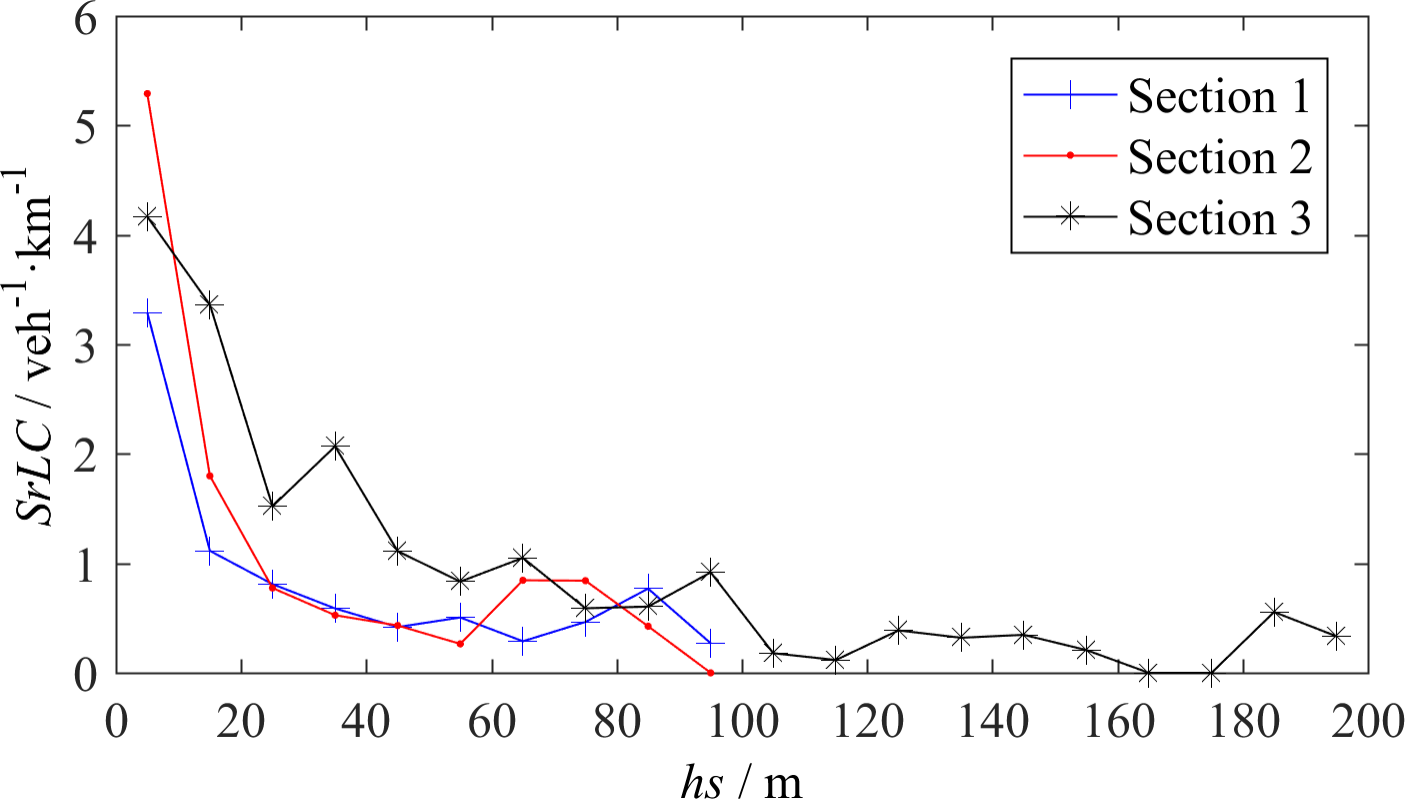
LC rate–space headway relationship.

Fig 6 reveals the relation between *SrLC* and space headway *hs*. Critical values of *hs* exist. When *hs* is less than the critical value, *SrLC* almost monotonically increases as *hs* decreases; when *hs* is larger than the critical value, the *SrLC* only slightly changes at a certain level. For S1 and S2, the critical *hs* ranges from 30~40 m; for S3, the critical *hs* is approximately 100 m. The difference of the critical *hs* among the three sections reflects the discrepancy between urban expressways and intercity highways. Vehicles on an intercity highway move faster than vehicles on an urban expressway and they are more sensitive to spacing; therefore, the *SrLC* of S3 for each spacing range is usually higher than the *SrLC* values of S1 and S2.

**Fig 7:**
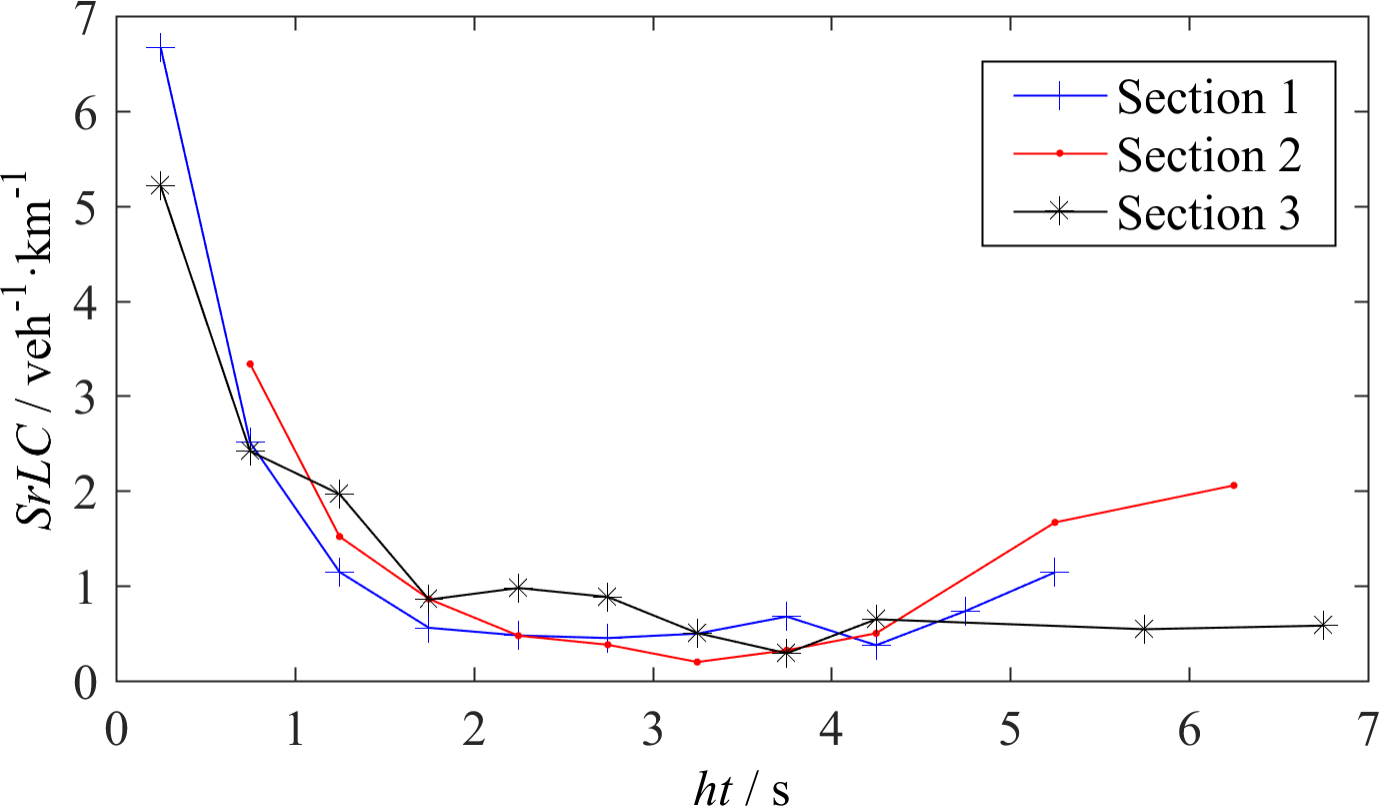
LC rate–time headway relationship.

The relationship between *SrLC* and the time headway *ht* is shown in Fig 7. The curves in Fig 7 have some similarities with the curves in Fig 6. The critical *ht* of approximately 2~3 s also exists. When *ht* is less than the critical value, *SrLC* monotonically increases as *ht* decreases. However, Fig 7 also shows some differences compared with Fig 6. First, in Fig 7, the three curves almost overlap when *ht* is less than the critical value, whereas the curve of S3 in Fig 6 is higher than the other two curves. This finding indicates that drivers on urban expressways or intercity highways, regardless of the range of vehicle speed, have the same sensitivity to the time headway when considering a LC. The time headway *ht* can be treated as an invariant to describe the effect of spacing on LC in any section. Second, for S1 and S2, the *SrLC* almost remains constant when *ht* is between 2 s and 4 s and continue to increase when *ht*>4 s, which reveals a tail-raising tendency. In Fig 6, the tail-raising tendency for S1 and S2 is not as significant. In our observations, the number of vehicles with *ht*>4 s is relatively small but the mandatory LCs maintain a certain amount, which cause a tail-raising tendency. Due to the lack of ramps at S3, this tendency is not observed on the corresponding curve.

Chang and Kao reported the similar effect of time headway on LC [17]. However, their results cannot be quantitatively compared with ours, because they simply used the number of lane changes. That is why we recommend to adopt *SrLC* and *TrLC*.

#### Effect of adjacent lanes

**Fig 8:**
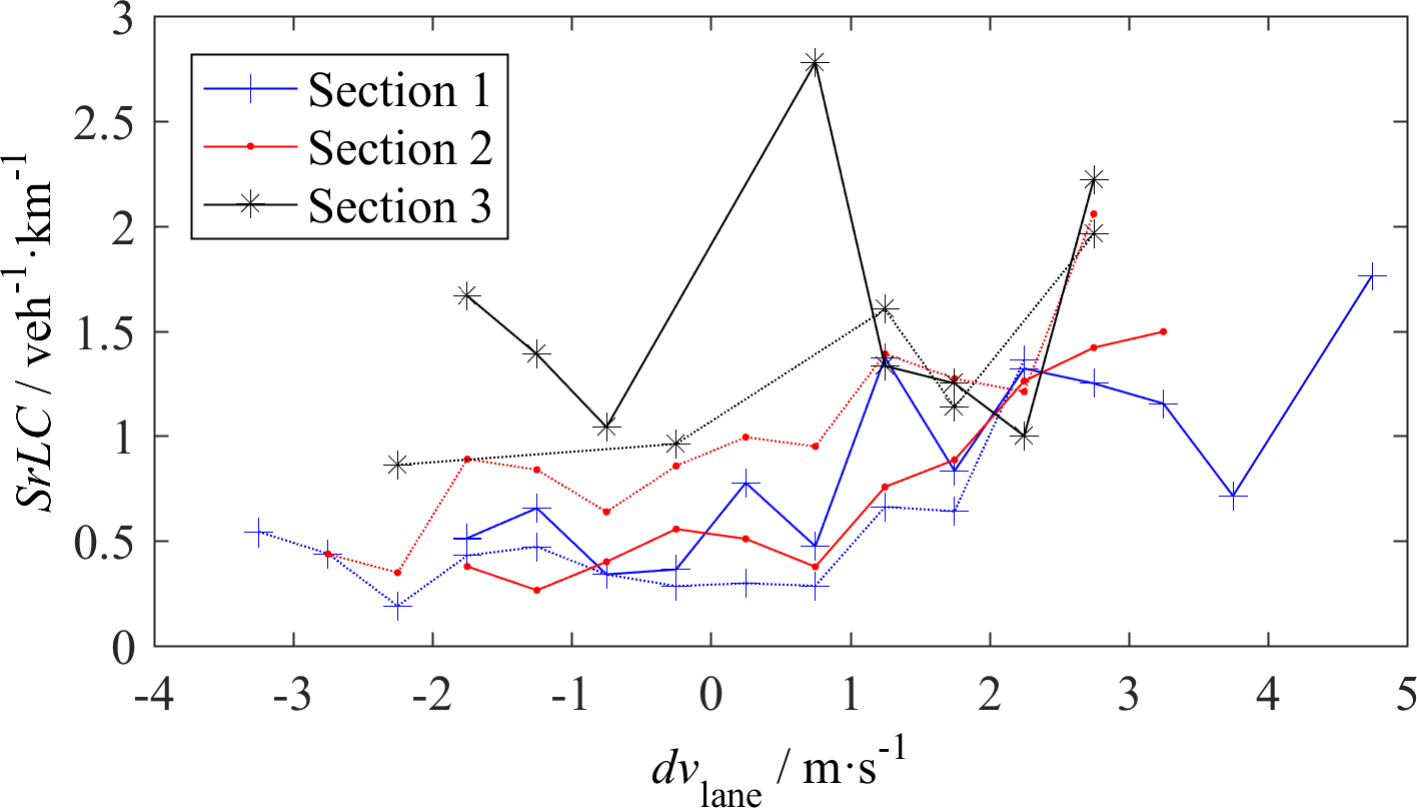
LC rate–speed difference across the lanes (*dv*_lane_) relationship, the solid lines represent *SrLC*_L_ and the dash lines represent *SrLC*_R_.

**Fig 9:**
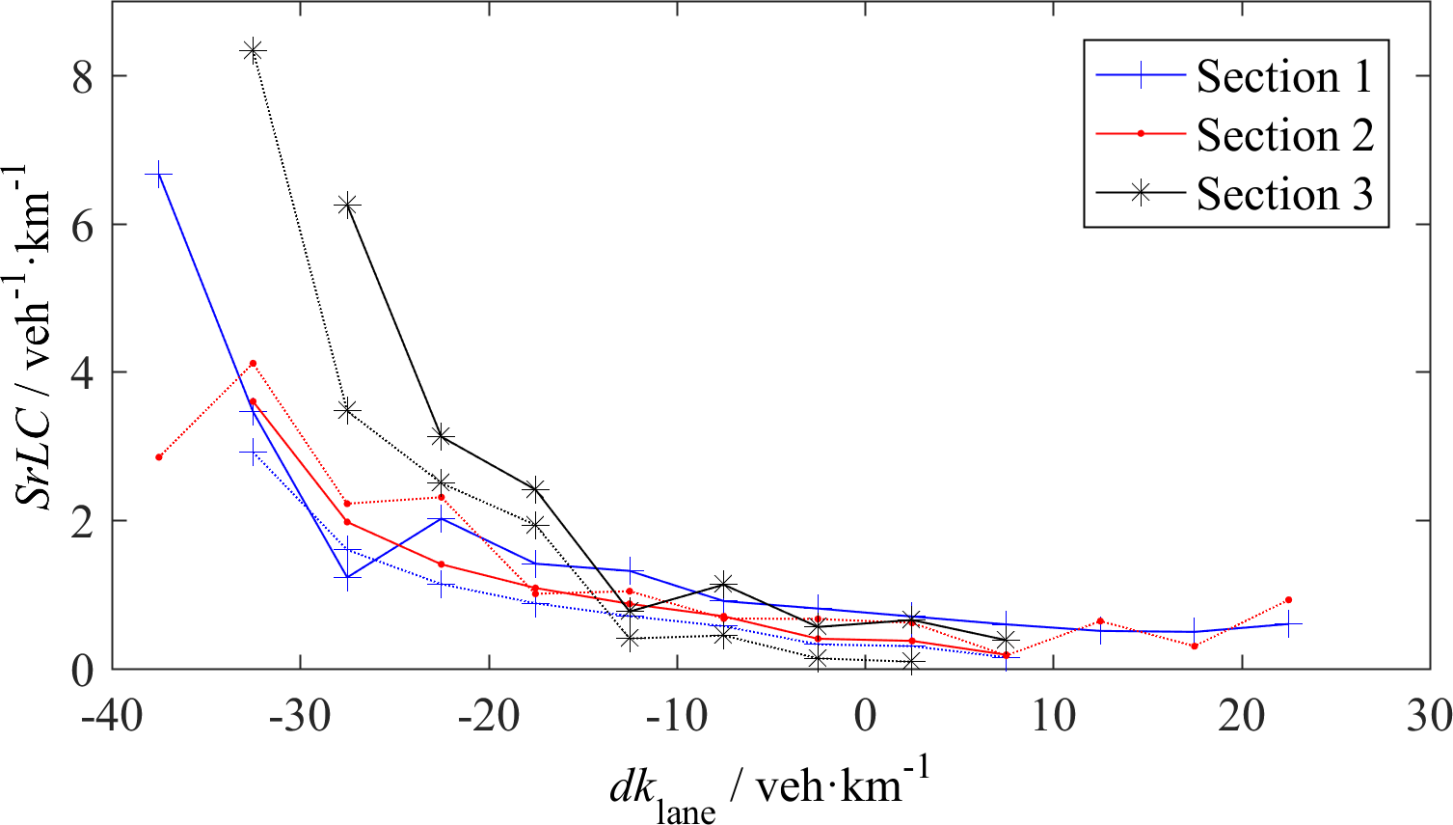
LC rate–density difference across the lanes (*dk*_lane_) relationship, the solid lines represent *SrLC*_L_ and the dashed lines represent *SrLC*_R_.

The speed difference and density difference between adjacent lanes, i.e., *dv*_lane_ and *dk*_lane_, are important factors that affect LC. Because each of the two middle lanes has a left adjacent lane and a right adjacent lane, when analyzing the effect of *dv*_lane_ and *dk*_lane_, we separately counted the *SrLC* changing into the left lane (denoted by *SrLC*_L_) and changing into the right lane (denoted by *SrLC*_R_). Figs 8 and Figs 9 show the effect of *dv*_lane_ and *dk*_lane_, respectively.

Although the *SrLC*-*dv*_lane_ relationship in Fig 8 is not as distinct as the *SrLC*-*dv* relationship, some valuable conclusions can be formed. At S1 & S2, the critical value of *dv*_lane_ is approximately 1 m/s instead of 0 m/s, i.e., when *dv*_lane_ is less than 1 m/s, *SrLC*_L_ and *SrLC*_R_ remain constant or slightly increase with *dv*_lane_. When *dv*_lane_ is sufficiently large, drivers on urban expressways are induced to increase the LC frequency. However, *SrLC*_R_ at S2 is an exception, which always increases with *dv*_lane_; even in the range of negative *dv*_lane_, its value is rather large. This finding reflects the strong desire for mandatory LC near an off-ramp that is located 250 m downstream. At S3, *SrLC*_L_ appears excessively high and irrelevant with *dv*_lane_, whereas *SrLC*_R_ linearly increases with *dv*_lane_, which may reveal different dominating incentives for changing to the left lane and changing to the right lane at S3. Chinese drivers on intercity highways tend to move as fast as they can; thus, they attempt to change to the left lane whenever possible.

Compared with *dv*_lane_, *dk*_lane_ has a distinct effect on LC, as shown in Fig 9. The density non-uniformity across the lanes is more likely to trigger LC than the speed non-uniformity. All *SrLC*s monotonically decrease as *dk*_lane_ increases. These reductions have steep slopes when *dk*_lane_<-10 veh/km, especially for S3, where vehicle speeds are high. When *dk*_lane_>-10 veh/km, its effect does not have apparent difference among the three sections, which indicates that a large *dk*_lane_ has a greater effect on fast vehicles than slow vehicles. As shown in Fig 8 and Fig 9, *SrLC*_L_ is larger than *SrLC*_R_ at S1 and S3, which reflects the attraction of the fast lane. At S2, *SrLC*_L_ is less than *SrLC*_R_, which reflects the attraction of the off-ramp.

**Fig 10:**
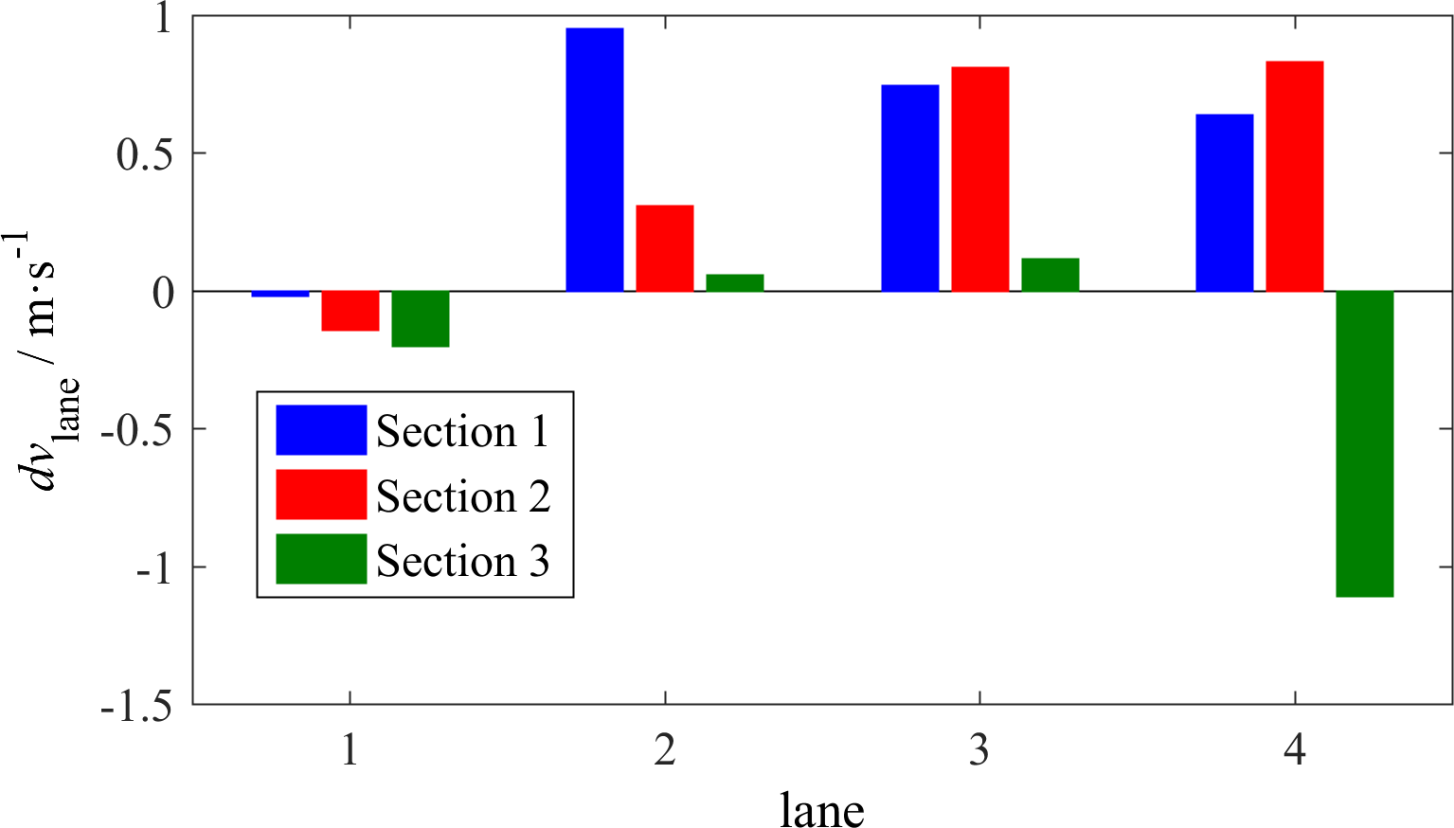
Average *dv*_lane_ that corresponds to LC vehicles in each lane.

**Fig 11:**
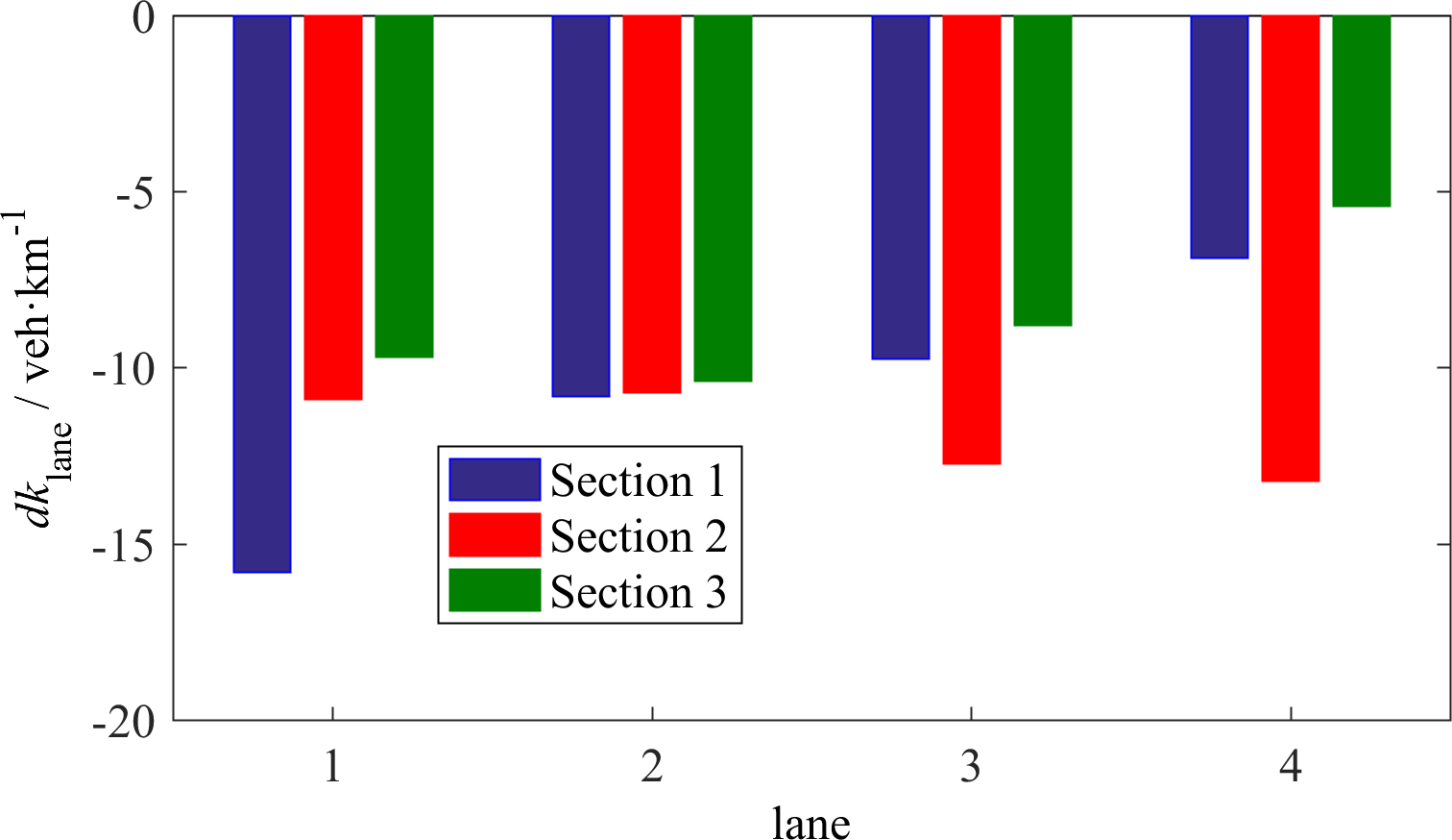
Average *dk*_lane_ that corresponds to LC vehicles in each lane.

Regarding different individual lanes, the effect of *dv*_lane_ or *dk*_lane_ differs, as shown in Figs 10 and 11. The bar charts were obtained as follows: For each of the LC vehicles, we calculated the average *dv*_lane_ or *dk*_lane_ between the target lane and the original lane before the LC occurred, and then counted the mean values of the results grouped by the original lanes. The mean values of *dv*_lane_ for each lane may be positive or negative, whereas all mean values of *dk*_lane_ have the same sign, i.e., negative. This finding indicates that the density of the adjacent lane is considered prior to the speed of this lane when a driver intends to change lanes. In Fig 11, the absolute values of average *dk*_lane_ at S1 decrease from L1 to L4, which reveals that the adjacent lane may be attractive to vehicles in the fast lanes when it is significantly vacant compared with the original lane. The average *dk*_lane_ at S3 has a similar tendency. However, the absolute values are smaller than the absolute values at S1, which proves that the faster a vehicle travels, the stronger is the desire to engage in LC. The average *dk*_lane_ at S2 has the opposite tendency. This finding reveals that upstream of an off-ramp, vehicles in the right lanes have a relatively low demand to change lanes unless the adjacent lane is vacant. Regarding the average *dv*_lane_, two thirds of the values are positive, which indicates that the majority of LC vehicles moved to a faster lane during these moments. It is noticeable that the average *dv*_lane_ on L4 at S3 has a large negative value, which provides evidence of aggressive driving. As previously mentioned, some cars move to L4 based on the availability of space without worrying about heavy vehicles, however, they would like to change back to the fast lane to avoid slow vehicles whenever L3 has the minimum required space for LC despite the average speed on L3. In addition, the small values of *dv*_lane_ on L2 and L3 at S3 support this explanation.

**Fig 12:**
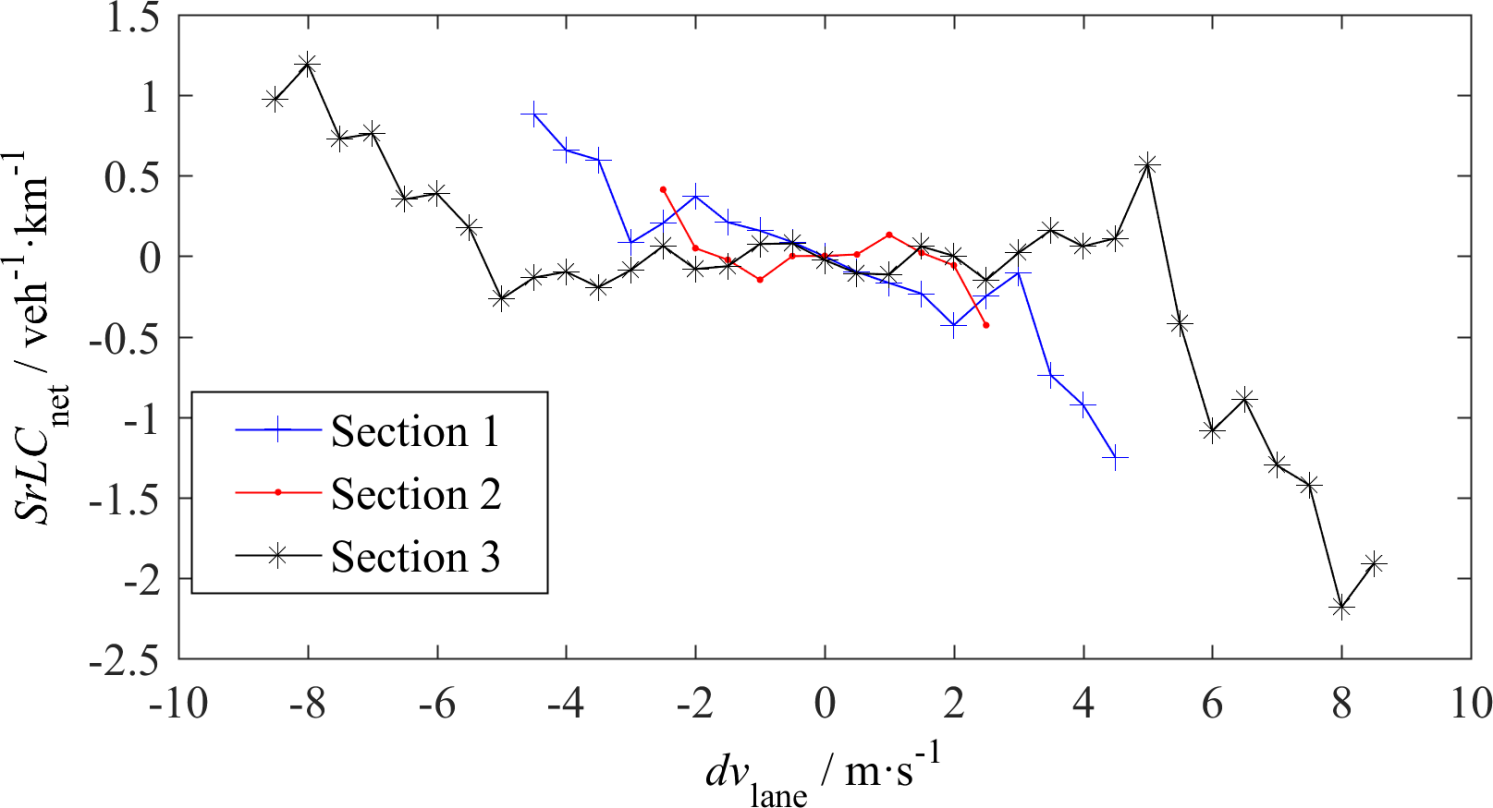
Net inflow LC rate—*dv*_lane_ relationship.

**Fig 13:**
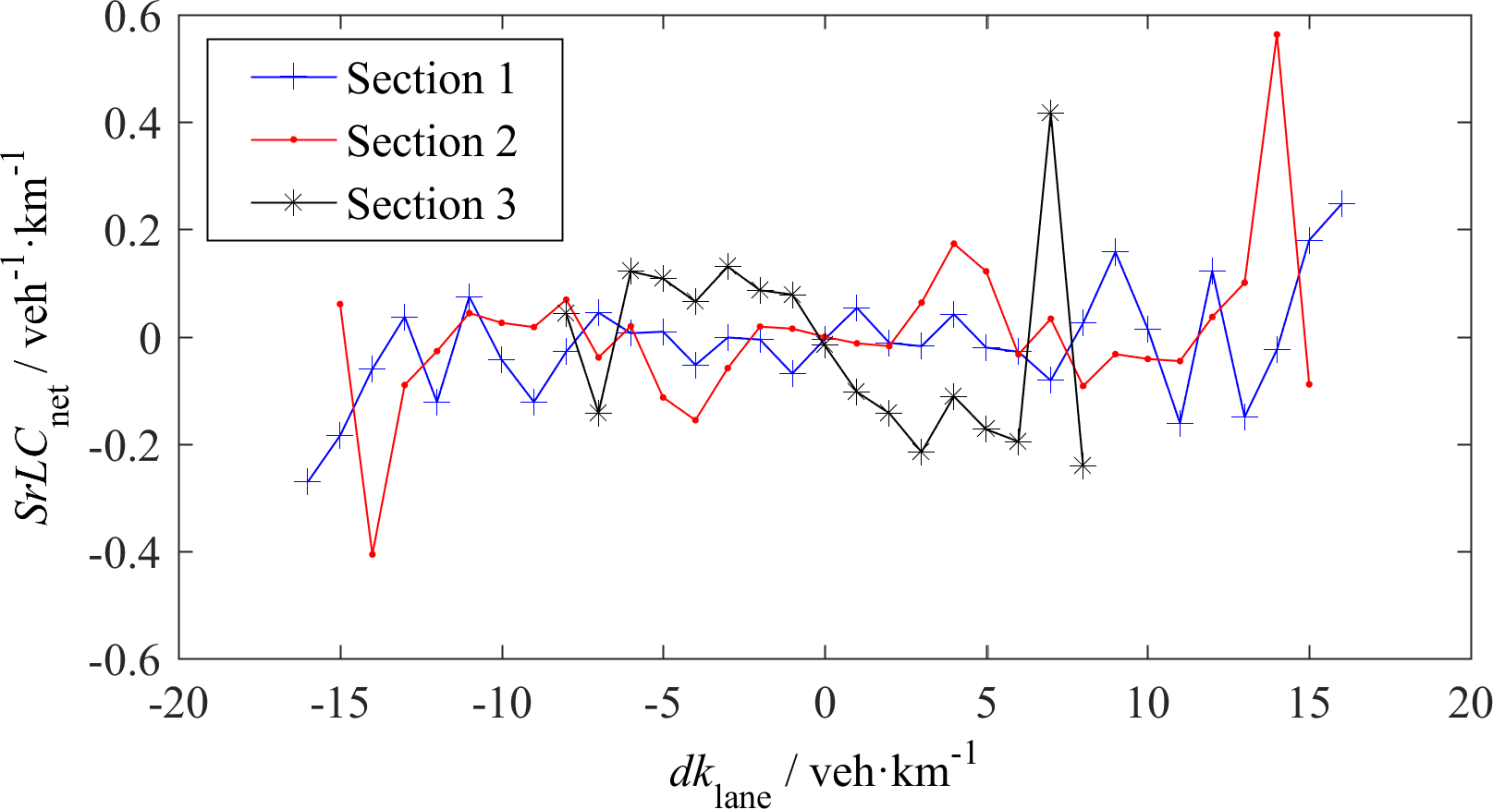
Net inflow LC rate -*dk*_lane_ relationship.

In the macroscopic LC model proposed by Laval and Leclercq [4, 6] and Ko et al. [13], the net inflow LC rate is a linear function of *dv*_lane_ and *dk*_lane_. Fig 12 and Fig 13 show the measured *SrLC*_net_ with respect to *dv*_lane_ and *dk*_lane_ respectively, where *SrLC*_net_ is the net inflow spatial LC rate. The curves were obtained as follows: First, a time window of 1 minute was set to count the net vehicle inflow (produces positive *SrLC*_net_) or outflow (produces negative *SrLC*_net_); second, we moved the time window with an interval of 1 s along the data sets to calculate the *SrLC*_net_, *dv*_lane_ and *dk*_lane_ for each lane within the time window; and last, all *SrLC*_net_ data were grouped into different ranges of *dv*_lane_ or *dk*_lane_, and the mean and median *SrLC*_net_ in each range were plotted. The difference between mean *SrLC*_net_ and median *SrLC*_net_ is minimal. Therefore, either parameter can represent the general trends of *SrLC*_net_ with *dv*_lane_ or *dk*_lane_.

*SrLC*_net_ is not a simple linear function of either *dv*_lane_ or of *dk*_lane_. In Fig 12, at S1, the relationship between *SrLC*_net_ and *dv*_lane_ is linear as some researchers have assumed. At S3, when the absolute value of *dv*_lane_ is larger than 5 m/s, *SrLC*_net_ linearly decreases with *dv*_lane_; otherwise, *SrLC*_net_ almost remains constant. This finding indicates that a small *dv*_lane_ does not cause LC across the lanes for free-flow traffic with high vehicle speeds. At S2, however, the linear relationship almost disappears, due to the mandatory LCs. In Fig 12, the absolute values of *SrLC*_net_ for a positive *dv*_lane_ are larger than the absolute values of *SrLC*_net_ for the corresponding negative *dv*_lane_, because the flow rates of fast lanes are higher than the flow rates of slow lanes. Fig 13 shows that *dk*_lane_ has no distinct impact on *SrLC*_net_.

#### Combined effect

These analyses have revealed the individual impact of multiple parameters on LC. We attempt to investigate their combined effects, i.e., how *dv*, *hs*, *dv*_lane_ and *dk*_lane_ cooperatively induce an LC and whether one of these parameters is dominant.

Table 5 presents the correlation matrices of the four variables, in which a weak correlation is observed. Based on the correlation matrix, a principal component analysis (PCA) was performed. Tables 6–8 lists the PCA results, where C1 to C4 denote the calculated principal components, PEV denotes the percentage of explained variance by each component and is an intuitive index of goodness of fit, CPEV denotes the cumulative PEV, and the coefficients are the respective correlation of the variables with the corresponding components. For instance, in Table 6, the first principal component C1 = 0.69*dv*-0.37*hs*-0.60*dv*_lane_+0.15*dk*_lane_, accounts for 33.88% of the variance in the data set, C2 to C4 account for 22.44%, 17.56% and 16.12% of the variance, and the first three components explain 83.88% of the variance in this data set. The four principal components represent certain patterns of the accomplished LC. The combined effect of the four parameters on LC is complicated compared with their individual effect.

**Table 5:** Correlation matrix of *dv*, *hs*, *dv*_lane_ and *dk*_lane_.

	S1				S2				S3			
	*dv*	*hs*	*dv*_lane_	*dk*_lane_	*dv*	*hs*	*dv*_lane_	*dk*_lane_	*dv*	*hs*	*dv*_lane_	*dk*_lane_
*dv*	1	-0.23	-0.26	0.03	1	0.01	-0.15	0.21	1	-0.37	-0.23	-0.20
*hs*	-0.23	1	0.01	0.23	0.01	1	-0.16	0.31	-0.37	1	0.08	0.17
*dv*_lane_	-0.26	0.01	1	-0.20	-0.15	-0.16	1	-0.21	-0.23	0.08	1	0.34
*dk*_lane_	0.03	0.23	-0.20	1	0.21	0.31	-0.21	1	-0.20	0.17	0.34	1

**Table 6:** PCA results of LC motivation for S1.

principal component	coefficient of				PEV	CPEV	LC pattern
	*dv*	*hs*	*dv*_lane_	*dk*_lane_			
C1	0.69	-0.37	-0.60	0.15	33.88	33.88	speed-sensitive
C2	-0.12	0.62	-0.34	0.69	32.44	66.32	space-sensitive
C3	0.26	-0.30	0.66	0.64	17.56	83.88	adjacent lane-attracting
C4	0.66	0.62	0.31	-0.29	16.12	100.00	current lane-extruding

**Table 7:** PCA results LC motivation for S2.

principal component	coefficient of				PEV	CPEV	LC pattern
	*dv*	*hs*	*dv*_lane_	*dk*_lane_			
C1	0.38	0.50	-0.49	0.61	38.82	38.82	space-sensitive
C2	0.77	-0.61	-0.14	-0.09	24.87	63.69	current lane-extruding
C3	0.34	0.17	0.86	0.34	20.62	84.31	adjacent lane-attracting
C4	-0.39	-0.59	-0.01	0.71	15.69	100.00	ramp-attracting

**Table 8:** PCA results LC motivation for S3.

principal component	coefficient of				PEV	CPEV	LC pattern
	*dv*	*hs*	*dv*_lane_	*dk*_lane_			
C1	0.55	-0.47	-0.47	-0.50	42.56	42.56	generally aggressive
C2	-0.36	0.61	-0.56	-0.43	25.66	68.22	extremely aggressive
C3	0.45	0.29	-0.49	0.69	17.45	85.67	adjacent lane-attracting
C4	0.61	0.57	0.46	-0.30	14.33	100.00	current lane-extruding

An extremely small PEV is not obtained; the smallest PEV is larger than 14%, which indicates that all types of LC patterns serve important roles in real traffic and should not be disregarded. At S1, C1 represents the speed-sensitive LC pattern because *dv* and *dv*_lane_ have dominant coefficients. For this LC pattern, a slow lead vehicle or adjacent fast lane is the main cause of LC. Similarly, other LC patterns can be defined, as listed in Tables 6–8. The speed-sensitive and space-sensitive LC patterns, and the adjacent lane-attracting and current lane-extruding LC pattern have similar proportions, i.e., 33.88% and 32.44%, and 17.56% and 16.12%, respectively. At S3, the first two LC patterns are defined based on the traffic video that shows aggressive driving circumstances. In the generally aggressive LC pattern, all variables have similar weights, which is unlike the other patterns that usually have two significant coefficients. LC vehicles are sensitive to each of the variables. In the extremely aggressive LC pattern, *hs* and *dv*_lane_ have a greater weight than the other variables, which indicate that some drivers changed lanes if they were near the lead vehicle and tended to change to the fast lane for as long a distance as possible. The two aggressive LC patterns account for more than 68% of the variance in this data set, which reveals the behaviors of aggressive drivers on intercity highway. The proportions of the other two LC patterns, i.e., C3 and C4, are similar to the proportions at S1. Due to the existence of mandatory LCs, the four patterns at S2 are not as distinguishable as the four patterns at S1 and S3. Compared with S1, the space-sensitive LC pattern increases to first place, and the speed-sensitive LC pattern is absent. In C4, the weight of *dv*_lane_ is negligible, which is unique in Tables 6–8. In the mandatory LCs, *dv*_lane_ is not considered. Therefore, C4 represents the ramp-attracting LC pattern. The PEV of C4 is approximately 16 percent. The percentage is a slightly lower than the proportion of the mandatory LC estimated from the data in Tables 3 and 4. Thus, the majority of the mandatory LCs belong to this pattern.

The positive and negative signs of the coefficients in the principal components are associated with the signs in Table 5, which indicates some features of LC. For example, *dv* and *hs* of LC vehicles have a weak negative correlation at S1 and S3. When the speed of the lead vehicle is significantly lower than the speed of the subject vehicle, the driver is likely to change lanes from a great distance. If the speed difference is small, the driver is likely to change lanes until they are close. This negative correlation between *dv* and *hs* does not exist for non-LC vehicles at S1 and S3, whose corresponding correlation coefficients are -0.05 and 0.21 respectively.

### Target lane choice

**Fig 14:**
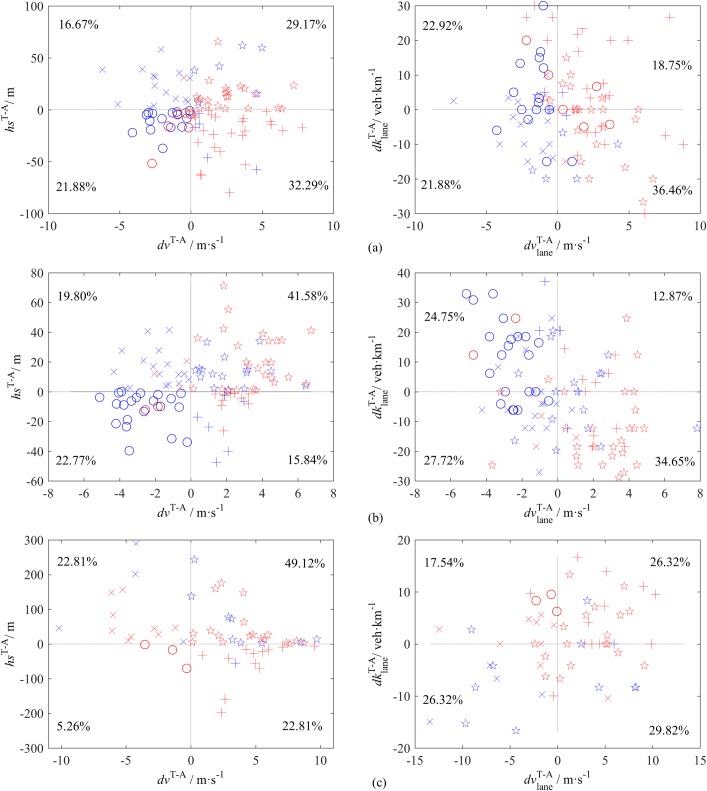
Differences in traffic condition between the target lane and the alternative lane. The red markers denote left-turning LC, and blue markers denote right-turning LC. The percentages represent the proportion of LC in each quadrant. The four types of markers indicate the quadrant in the *hs*^T-A^—*dv*^T-A^ plane associated with each LC, i.e., ☆: 1st quadrant, x: 2nd quadrant, o: 3rd quadrant and +: 4th quadrant. (a) S1. (b) S2. (c) S3.

A vehicle that travels in the middle lanes has two options when changing lanes, namely, the target lane that is chosen and the alternative lane that is not chosen. In this subsection, we investigate the effect of target lane choice for the LC vehicles in L2 and L3. The minimum values of *hs* and *ht* between the subject vehicles and their lead and following vehicles in the target lanes, as listed in Table 9, were jointly considered as the lower limitations for the feasibility of LC to eliminate the unavailable alternative lanes. Then, the difference in the traffic condition between the target lane and the alternative lane were plotted in the *hs*^T-A^—*dv*^T-A^ plane and the $dk_{lane}^{T-A}$ - $dv_{lane}^{T-A}$ plane, respectively, as shown in Fig 14, where the superscript T-A denotes the value of the target lane minus the value of the alternative lane.

**Table 9:** Lower limitations of *hs* and *ht* for the feasibility of LC.

	lead vehicle		following vehicle	
	*hs/*m	*ht/*s	*hs/*m	*ht/*s
S1	5.80	0.34	5.11	0.45
S2	7.11	0.50	5.45	0.58
S3	12.95	0.52	8.12	0.32

The optimal choice is supposed to be in the first quadrant of *hs*^T-A^—*dv*^T-A^ plane and the fourth quadrant of $dk_{lane}^{T-A}$ - $dv_{lane}^{T-A}$ plane, whereas the worst choice is in the third quadrant of *hs*^T-A^—*dv*^T-A^ plane and the second quadrant of $dk_{lane}^{T-A}$ - $dv_{lane}^{T-A}$ plane. The proportions of the worst choices, i.e., the marker ‘o’ in the second quadrant of the $dk_{lane}^{T-A}$ - $dv_{lane}^{T-A}$ plane, are 10.42% at S1, 7.46% at S2 and 5.26% at S3. They primarily consist of right-turning LCs at S1 and S2 (80% and 86.67%, respectively), and left-turning LCs at S3 (100%). These results reveal the attractiveness of ramps on urban expressways and the fast lanes on intercity highways.

Fig 14 also shows that the target lane choice is determined by the combination of various factors. Similarly, PCA can be performed to identify the patterns of choosing the target lane. Similar results as those in Tables 6–8 are obtained, however, the CPEVs of the first two components are close to 90%. These results indicate the high consistency in target lane choice for drivers after deciding to change lanes.

### Impact on traffic

#### Impact on time headway

**Fig 15:**
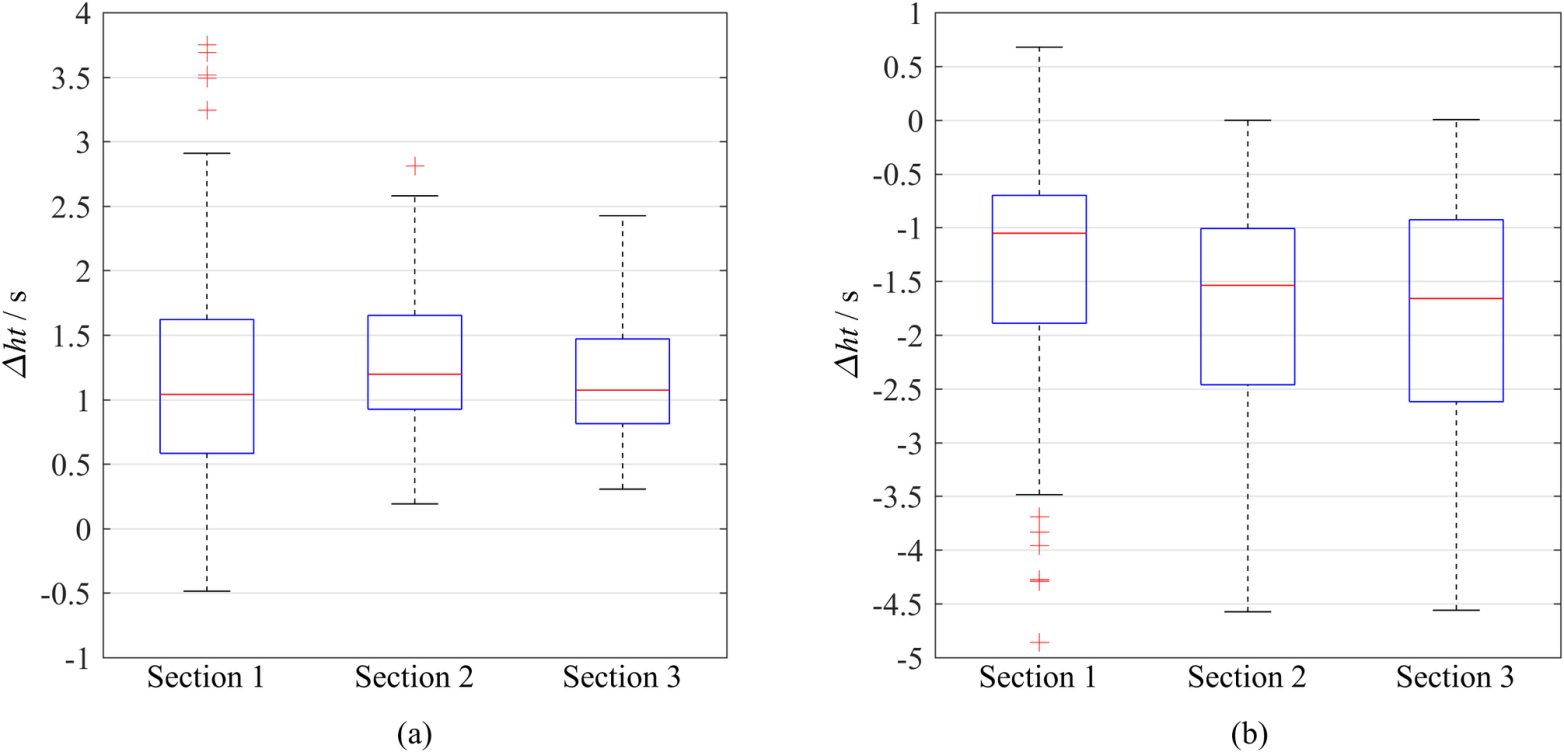
Boxplots of the changes in time headway for the immediate followers before and after LCs. (a) In the original lane. (b) In the target lane.

An LC increases the time headway *ht* of the immediate follower in the original lane and decreases the *ht* of the immediate followerin the target lane. Fig 15 shows the *ht* changes (i.e., *ht* after LC minus *ht* before LC) of the immediate followers. In the original lane, the median increases in the *ht* are slightly larger than 1 s, which are similar for the three sections because the rear vehicle in the same lane is seldom considered during an LC process. In the target lane, the median decreases in the *ht* of the three sections differ: the higher is the speed and the smoother is the traffic, the larger is the decrease in *ht*. For example, at S3, the median *ht* of the immediate follower in the target lane decreases from 2.81 s to 1.17 s, which is not sufficiently safe for driving in this section, where the average speed is 26.13 m/s.

The safe *ht* is slightly lower than 2 s, for example, German authorities suggest 1.8 s for the safe time headway [35]. Therefore, an *ht* less than 1 s is considered to be a dangerous behavior. Table 10 compares the percentages of vehicles with *ht*<1 s in total vehicles and LC vehicles. The latter percentages are twice even thrice the former percentages. To test whether there was significant difference in *ht* of LC vehicles and non-LC vehicles, a non-parametric method of analysis of variance (ANOVA), i.e., Kruskal-Wallis test was applied because *ht* does not obey the normal distribution. The result shows the p-values are almost zero for S1, S2 and S3, which demonstrates the significant difference in *ht* of LC vehicles and non-LC vehicles. Therefore, this type of LC style remarkably adds risk to traffic.

**Table 10:** LC’s influence on *ht*.

Section	Percentage of *ht*<1 s in total vehicles	Percentage of *ht*<1 s in LC vehicles
1	24.64%	52.06%
2	15.55%	46.62%
3	15.54%	41.76%

#### Impact on local speed

To evaluate the LC’s impact on a local site, we define the local speed change *dv*_local_ as

$$dv_{\mathrm{local}}={\bar{v}}_{i}-{\bar{v}}_{-10},$$ (6)

where ${\bar{v}}_{-10}$ is the average speed in the study lane within 10 s before LC, and ${\bar{v}}_{i}$ is the average speed in the same lane within the *i-*th s after LC. According to the variation in *dv*_local_ (i.e., the variation in ${\bar{v}}_{i}$) with time, we can determine the duration of the influence of an LC at the local site. To prevent the interaction of two or more LCs, if another LC occurs within the time period when calculating *dv*_local_, this data point will be omitted.

**Fig 16:**
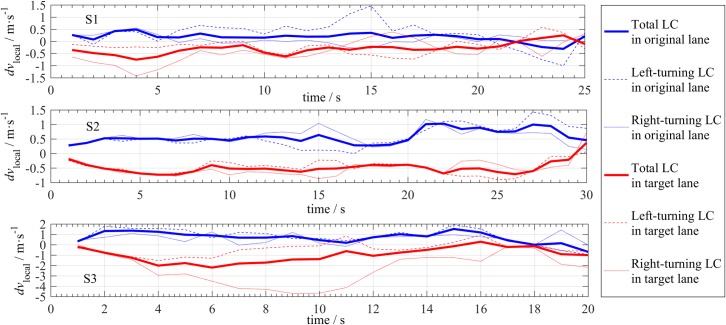
Trends of the local speed after LCs.

For each individual LC, the value of *dv*_local_ varies over a wide range due to the randomness of traffic. Therefore, we only investigate the trend of *dv*_local_. Fig 16 shows the variation of average *dv*_local_ with time in the original lane and the target lane. LC causes the speed in the original lane to increase (*dv*_local_>0) and cause the speed in the target lane to decrease (*dv*_local_<0), and the absolute value of the latter is larger than the absolute value of the former. However, this speed increase or decrease is not large. The maximum absolute value of *dv*_local_ in Fig 16 is usually less than 10% of the average speeds of the corresponding road sections, which implies that most of the followers are not willing to decelerate to achieve adequate spacing. In addition, for the target lane, the speed decrease caused by right-turning LC is larger than the speed decrease caused by left-turning LC, which indicates that the LC causes more speed loss for the slow lane than the fast lane.

As shown in Fig 16, the values of *dv*_local_ return to 0 after 15 to 30 s, i.e., the influence of an LC on the local site lasts approximately 15 to 30 s, depending on the specific site and traffic condition. The smoother is the traffic, the shorter is the duration of the influence, because *dv*_local_ at S3 returns to zero in less than 20 s after LC, which is shorter than *dv*_local_ at S1 and S2. In free flow, the duration of the LC’s perturbation is greater than 15 s at the local site, which is not short in real traffic. The LC’s influence on local speed is a two-stage process, namely, influence increasing and influence declining. The duration of the former stage for the target lane is approximately 5 s, which is slightly longer than the duration of the former stage for the original lane (approximately 3 s). Thus, the influence of LC rapidly increases, and slowly declines. The local disturbance requires a certain time period to dissipate.

Some of these conclusions are not very clear at S2, which may be attributed to the off-ramp. Frequent LCs occur prior to this off-ramp, which creates complicated situation.

## Conclusion

This empirical study explored LC behavior in aggressive driving circumstances. The main characteristics of this kind of LC are as follows:

1. Spatial LC rate *SrLC* and temporal LC rate *TrLC* quantitatively demonstrate the high LC frequency on urban expressways and intercity highways in China, which is twice the rates in other studies. On these freeways, LC is a transient behavior that randomly occurs for a better ambient condition. Therefore, the net inflow or outflow across lanes is not highly correlated to the speed difference or density difference between adjacent lanes, especially for the density difference.
2. Aggressive drivers change lanes whenever they want regardless of the speed. Thus, the *SrLC* is more relevant to speed in high-speed driving, whereas the *TrLC* always increases with speed.
3. On intercity highways, the mixture of different types of vehicles increases the LC rate. This mixture also leads to the attracting and anchoring effect of the fast lane, especially the leftmost lane.
4. On urban expressways, the attraction of off-ramps and the repulsion of on-ramps increase the LC rates, especially LC in the lane with worse ambient conditions.

We analyzed the motivation of LC in aspects such as speed, spacing, the conditions in the current lane and the adjacent lane, and provided the relationship between the LC rate and these parameters, especially some of their critical values. Given that an LC is the result of a combined effect, we summarized several LC patterns and their percentages of explained variance using PCA.

LC has severe impacts on traffic flow in the target lane. LC usually remarkably decreases the time headway of the subject vehicle and the immediate follower, which increases the risk of high-speed car-following. At the local site of LC, the duration of the LC’s influence ranges from 15 to 30 s, which rapidly increases and slowly declines.

This study provides quantitative data for modeling and validation. We believe that a suitable LC model should simulate the following three items: (1) LC rate, (2) impact on the target lane’s traffic, and (3) the percentage of high-speed car-following. We hope that the insightful knowledge of LC in manual driving can aid in the strategy design of automatic driving.

Future improvements of this study are necessary. These conclusions are formed from three half-hour observations; however, they are consistent with our driving experience. Therefore, additional LC data in various traffic conditions are needed despite the exhausting process of data collecting. The abortive attempt to change lanes can also affect traffic, which warrants additional studies. Constrained by the data collection method, only the disturbanceto local traffic was explored, and a new data collection method is needed to obtain LC data for long road sections.

## Supporting information

S1 Dataset The raw data employed in this paper. (RAR)

S1 Video A typical scenario at section 3. (MP4)
